# MULTIPLE SEVERE WEATHER EVENTS: ARE OLDER ADULTS MORE VULNERABLE?

**DOI:** 10.1093/geroni/igad104.0501

**Published:** 2023-12-21

**Authors:** Katie Cherry, Matthew Calamia, Emily Elliott, Piper Bordes, Laura Sampson, Sandro Galea

**Affiliations:** Louisiana State University, Baton Rouge, Louisiana, United States; Louisiana State University, Baton Rouge, Louisiana, United States; Louisiana State University, Baton Rouge, Louisiana, United States; Louisiana State University, Baton Rouge, Louisiana, United States; Harvard University, Boston, Massachusetts, United States; Boston University, Boston, Massachusetts, United States

## Abstract

Hurricanes and floods have psychosocial consequences for directly and indirectly affected individuals. In August of 2016, historic flooding in Baton Rouge, Louisiana resulted in catastrophic damage across a 22-parish (county) region only 11 years after the 2005 Hurricanes Katrina and Rita devastated the US Gulf Coast. In this study, we compared three groups of mostly middle-aged and older adults on the SF-36 Health Survey Questionnaire: (1) non-flooded (controls), (2) single disaster (flooded in 2016), and (3) double disaster (flooded in 2005 and again in 2016). Results indicated that the groups did not differ in self-reported physical health, although the single and double disaster groups reported worse mental health than did the controls. Correlation analyses revealed that age was positively associated with mental health. State hope was positively associated with both physical and mental health. Implications of these findings for reducing vulnerabilities after multiple disaster exposures are considered.

